# Neutrophil Elastase Causes Tissue Damage That Decreases Host Tolerance to Lung Infection with *Burkholderia* Species

**DOI:** 10.1371/journal.ppat.1004327

**Published:** 2014-08-28

**Authors:** Manoranjan Sahoo, Laura del Barrio, Mark A. Miller, Fabio Re

**Affiliations:** 1 Department of Microbiology and Immunology, Rosalind Franklin University of Medicine and Science, North Chicago, Illinois, United States of America; 2 Department of Microbiology, Immunology, and Biochemistry University of Tennessee Health Science Center, Memphis, Tennessee, United States of America; Emory University School of Medicine, United States of America

## Abstract

Two distinct defense strategies can protect the host from infection: resistance is the ability to destroy the infectious agent, and tolerance is the ability to withstand infection by minimizing the negative impact it has on the host's health without directly affecting pathogen burden. *Burkholderia pseudomallei* is a Gram-negative bacterium that infects macrophages and causes melioidosis. We have recently shown that inflammasome-triggered pyroptosis and IL-18 are equally important for resistance to *B. pseudomallei*, whereas IL-1β is deleterious. Here we show that the detrimental role of IL-1β during infection with *B. pseudomallei* (and closely related *B. thailandensis*) is due to excessive recruitment of neutrophils to the lung and consequent tissue damage. Mice deficient in the potentially damaging enzyme neutrophil elastase were less susceptible than the wild type C57BL/6J mice to infection, although the bacterial burdens in organs and the extent of inflammation were comparable between C57BL/6J and elastase-deficient mice. In contrast, lung tissue damage and vascular leakage were drastically reduced in elastase-deficient mice compared to controls. Bradykinin levels were higher in C57BL/6 than in elastase-deficient mice; administration of a bradykinin antagonist protected mice from infection, suggesting that increased vascular permeability mediated by bradykinin is one of the mechanisms through which elastase decreases host tolerance to melioidosis. Collectively, these results demonstrate that absence of neutrophil elastase increases host tolerance, rather than resistance, to infection by minimizing host tissue damage.

## Author Summary

Two distinct defense strategies can protect the host from infection: resistance is the ability to destroy the infectious agent, and tolerance is the ability to withstand infection by minimizing the negative impact it has on the host's health without directly affecting pathogen burden. *Burkholderia pseudomallei*, the causative agent of melioidosis, is a Gram-negative intracellular bacteria that is categorized as a potential bioterrorism agent. Using murine models, we previously demonstrated that during *B. pseudomallei* infection, production of IL-1β is deleterious as it recruited excessive neutrophils to the site of infection. In the present work, we focused on the detrimental role of neutrophils during infection with *B. pseudomallei* and *B. thailandensis*. Here, we demonstrate that the excessive recruitment of neutrophils to the site of infection causes tissue damage because of release of the protease elastase. Mice lacking neutrophil elastase have increased survival even though they carry an equal amount of bacteria in their organs as compared to the wild-type C57BL/6J. Thus, neutrophil elastase is a host defense mechanism that causes tissue damage and reduces host tolerance to infection.

## Introduction

Although the inflammatory response elicited by infection is highly effective against microbes, it is also greatly pathogenic and, in extreme cases, the damage caused to host tissues becomes the main source of morbidity and mortality associated with the disease [1]. After infection has occurred, the host can protect itself using mainly two distinct strategies: resistance or tolerance (reviewed in [2]–[4]. Resistance relates to the host's ability to destroy and eliminate the infectious agent. In contrast, tolerance does not directly affect pathogen burden, but rather it is a measure of the host's ability to withstand the presence of the infectious agent by minimizing the negative impact it has on the host's health. Much of what is known about the protective response to infection concerns resistance mechanisms and clearly falls in the realm of immunology. In contrast, the phenomenon of tolerance to infection in animals has remained largely unexplored, and only recently has begun to attract the attention of immunologists and microbiologists.

*Burkholderia pseudomallei* is a Gram-negative flagellate bacterium that causes melioidosis, a disease endemic to South-East Asia and other tropical regions [5]. Because melioidosis carries a high fatality rate, *B. pseudomallei* is classified as category B potential bioterrorism agent by the CDC and NIAID. *B. pseudomallei* infection can be contracted through ingestion, inhalation, or subcutaneous inoculation, and can lead to a broad-spectrum of disease forms including pneumonia, septicemia, and organ abscesses. Following infection of macrophages and other non-phagocytic cell types, *B. pseudomallei* is able to escape the phagosome and replicate in the host cell cytoplasm. Using a murine model of melioidosis, we have recently shown that the inflammasome component NLRP3 mediates caspase-1-dependent production of IL-1β and IL-18 while NLRC4 mediates pyroptosis [6]. While IL-18 and pyroptosis were equally important for protection from melioidosis, IL-1β was found to have a deleterious role. Recent studies have confirmed the deleterious role of IL-1β in melioidosis [7], [8].

To understand the reason for the detrimental effect of IL-1β during melioidosis, we have now focused our attention on the role of neutrophils and the potentially damaging enzyme neutrophil elastase (NE). Our results show that NE-deficient mice had an improved survival compared to C57BL/6J mice infected with *B. thailandensis* or *B. pseudomallei.* Surprisingly, bacterial burdens were comparable between C57BL/6J and NE-deficient mice, but lung tissue damage was reduced in NE-deficient mice. Taken together, our results suggest that NE is a host effector mechanism that negatively impacts host tolerance to infection with *Burkholderia* species.

## Results

### IL-1β Is Deleterious during Infection with *B. thailandensis* and *B. pseudomallei*

Although not pathogenic to humans, *B. thailandensis* causes morbidity and mortality in mice, shares several of the virulence factors of *B. pseudomallei*, and is often used as a model for melioidosis [9], [10]. Confirming what we observed during infection with *B. pseudomallei* [6], production of IL-1β following infection with *B. thailandensis* was dependent on NLRP3, ASC, and caspase-1 while pyropotosis was dependent on NLRC4 (supplementary figure S1A–D).

**Figure 1:**
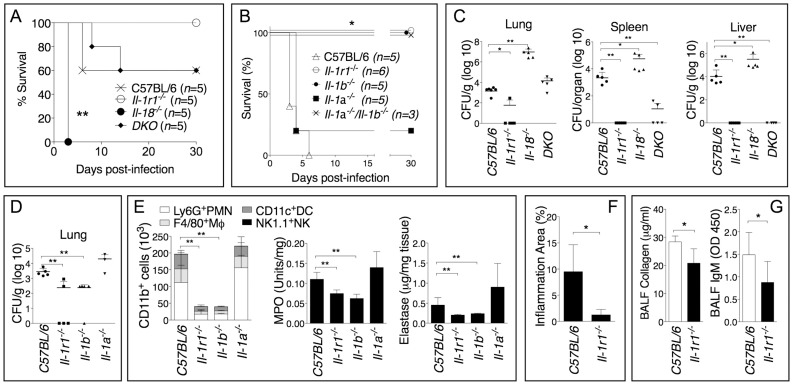
Deleterious role of IL-1β during infection with *B. thailandensis*. Mice were infected intranasaly with *B. thailandensis* (A, 5×10^4^ CFU or B, 5×10^5^ CFU) and their survival was monitored. (C, D) Mice infected with *B. thailandensis* (5×10^5^ CFU) were sacrificed 72 h post-infection and the organs bacterial burden in the indicated tissues was measured. (E) Flow cytometric analysis of BAL cells and MPO and elastase activity levels in the lung homogenates obtained from the indicated mouse strains at 72 h p.i. (*n* = 5 for all except *Il-1a^−/−^ n* = 3). (F) The total area of the inflammatory nodules of lung sections stained with H&E was measured and expressed as percentage of the total lung lobe area (*n* = 5). (G) IgM and collagen were measured in the BALF collected 72 h p.i. from mice (*n* = 5) infected with *B. thailandensis* (5×10^5^ CFU). Data are expressed as mean + S.D. *p<0.05, **p<0.01. (A, B) log rank Kaplan-Meier test, (C–G) Mann-Whitney U test. DKO is *Il-1r1^−/−^/Il-18^−/−^*double deficient mouse. One representative experiment of three (A–C, F, G) or one (D–E) is shown.

Similar to our [6] and others [11] studies with *B. pseudomallei*, IL-18-deficient mice were more susceptible than C57BL/6J wild type mice to *B. thailandensis* infection (fig. 1A) and had higher bacteria burdens in organs (fig. 1C). In contrast, mice deficient in IL-1 receptor (*Il-1r1^−/−^*) were more resistant to infection with *B. thailandensis* (fig. 1B) and showed reduced organ bacteria burdens (fig. 1C). Mice deficient in both IL-18 and IL-1RI (DKO) were as susceptible as C57BL/6J, suggesting that the deleterious role of IL-1β counteracts the protective role of IL-18. As shown in figure 1B and D, the IL-1RI-mediated deleterious role during *B. thailandensis* infection was due to the action of IL-1β, not IL-1α, although both cytokines are produced during infection [6]. The increased survival and lower bacterial burden observed in *Il1-r1^−/−^* mice correlated with reduced neutrophil influx in the alveolar spaces, reduced levels of myeloperoxidase and elastase in lung homogenates (fig. 1E), and lower inflammation in the lung (fig. 1F), suggesting that neutrophil recruitment to the lungs is as deleterious in this infection model as it is during *B. pseudomallei* infection [6]. These results support the validity of *B. thailandensis* as a model to study the role of IL-1 and neutrophils in melioidosis. It is well established that activated neutrophils release proteases and toxic radicals that may cause immunopathology and damage to host tissue. In fact, reduced numbers of neutrophils in the BALF of *Il1-r1^−/−^* mice correlated with decreased host tissue damage as measured by lower amount of collagen and IgM in the BALF of these mice (fig. 1G).

### Neutrophil Elastase Decreases Host Tolerance to Infection with *B. thailandensis* and *B. pseudomallei*

**Figure 2:**
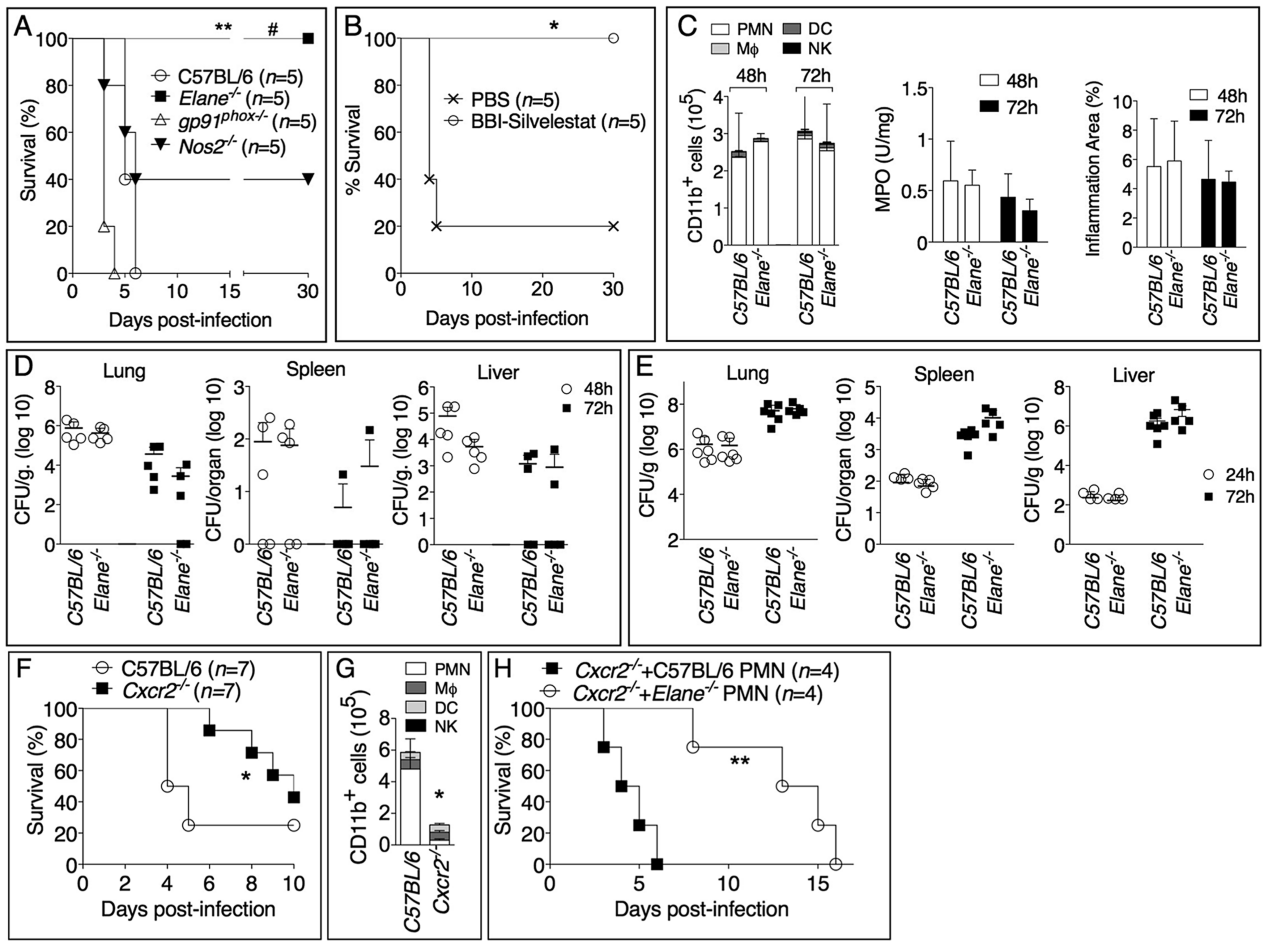
Neutrophil elastase reduces host tolerance to infection with *B. thailandensis*. (A) Mice were intranasaly infected with 5×10^5^ CFU *B. thailandensis* and their survival was monitored. (B) Elastase inhibitor sivelestat (125 µg/mouse, i.n.) and serine protease inhibitor BBI (3 mg/mouse, oral gavage) were administered on day 1, 2, 3 p.i. to C57BL/6 intranasaly infected with 1×10^5^ CFU *B. thailandensis*. (C) Flow cytometric analysis of BAL cells, levels of MPO activity in lung homogenates, and total area of the inflammatory nodules for mice infected with 5×10^5^ CFU *B. thailandensis* (*n* = 5). (D, E) Organs bacterial burden in the indicated tissues of mice infected with 5×10^5^ CFU (D) or 10^6^ CFU (E) *B. thailandensis*. (F) Wild type and *Cxcr2^−/−^* mice (*n* = 7, pool of two experiments) were intranasaly infected with 2×10^5^ CFU *B. thailandensis* and survival and BALF myeloid cells composition (G) (*n* = 4 C57BL/6J, *n* = 5 *Cxcr2^−/−^*) were measured. (H) *Cxcr2^−/−^* mice (*n* = 4) were intranasaly infected with 2×10^5^ CFU *B. thailandensis* and their survival was monitored. C57BL/6J or *Elane^−/−^* neutrophils (1.5×10^6^) were adoptively transferred by intravenous injection 24 and 96 hours p.i. Data are expressed as mean +S.D.*p<0.05, **p<0.01. # *p*<0.05 *Elane^−/−^* respect to *Nos2^−/−^*.(A, B, F, H) log rank Kaplan-Meier test, (C, D, E, G) Mann-Whitney U test. One representative experiment of three (A, C, D, E, G) or two (B, F, H) is shown.

To determine which of the potentially damaging effector mechanisms account for the deleterious role of neutrophils during infection with *B. thailandensis* we analyzed mice that lack expression of the ROS-generating enzyme NADPH oxidase (gp91^phox^), the inducible nitric oxidase (Nos2), or the NE (Elane). Previous works have shown that *gp91^phox−/−^* mice are more susceptible while *Nos2^−/−^* mice are more resistant to melioidosis [12], [13]. In order to detect a protective effect in these mouse strains, we infected them with a dose of *B. thailandensis* that was lethal to C57BL/6J mice (fig. 2A). At this dose, the survival of *gp91^phox−/−^* and *Nos2^−/−^* mice was not significantly different from that of C57BL/6J mice. In contrast, *Elane^−/−^* mice were completely protected from mortality. Although some level of protection was observed in *Nos2^−/−^* mice, in agreement with previous work [12], their survival was significantly lower (*p*<0.05) than that of *Elane^−/−^* mice suggesting that the deleterious role of NE is much more significant than that of iNOS. Bacteria could not be recovered from organs of *Elane^−/−^* mice that survived infection. Combined administration of sivelestat, a selective NE inhibitor, and the serine protease inhibitor BBI to infected C57BL/6J mice rescued their survival (fig. 2B), reinforcing the notion that NE activity is detrimental in melioidosis. Neutrophil recruitment to the lung and alveolar spaces and the overall extent of inflammation were not affected by the absence of NE (fig. 2C and supplementary figure S2). Quite surprisingly, C57BL/6J and *Elane^−/−^* mice had comparable bacterial burdens in their organs (fig. 2D). This was true even when mice were infected with a dose of bacteria that was lethal for both strains (fig. 2E). Thus, the higher health of *Elane^−/−^* mice, despite bacteria burdens similar to those of C57BL/6J mice, indicates that the absence of NE increases tolerance rather than resistance to *B. thailandensis*. It is worth noting that the previously reported protection of *Nos2^−/−^* mice was associated with decreased bacterial burdens [12] suggesting a resistance mechanism. In mammals, several genes encode for elastases, and the matrix metalloprotease MMP12 is the main elastase expressed in macrophages, a cell type that is infected by *B. thailandensis* and plays a prominent role in protection from melioidosis [14]. Interestingly, *Mmp12^−/−^* mice were not protected from infection with *B. thailandensis* (supplementary fig. S3).

To confirm the deleterious role of neutrophils during *B. thailandensis* infection, we performed parallel studies in mice deficient in expression of CXCR2, the chemokine receptor most critical for neutrophil recruitment to infection sites. As expected, neutrophil recruitment to the alveolar spaces was markedly reduced in *Cxcr2^−/−^* mice compared to C57BL/6J mice following infection with *B. thailandensis* (fig. 2G). *Cxcr2^−/−^* mice were also significantly less susceptible than C57BL/6J mice when infected with a dose that killed 72% of C57BL/6J mice (fig. 2F). This is a remarkable result considering that absence of CXCR2 is known to negatively impact the overall health status of the mice, which in fact appear runt and are more susceptible to several bacterial infections [15]–[17]. These findings were in agreement with our and other's previous observations that neutrophils are not particularly effective at controlling *B. thailandensis* or *B. pseudomallei* [6], [18]–[20], although a protective effect for these cells has also been reported [21]. Because of the defective neutrophil chemotaxis, *Cxcr2^−/−^* mice provided a fitting model to test the role of NE with the prediction that C57BL/6J or *Elane^−/−^* neutrophils, adoptively transferred into *Cxcr2^−/−^* mice, would be able to be recruited to the lung, following infection with *B. thailandensis*, and exert their deleterious effects. Indeed, as shown in figure 2H, transfer of C57BL/6J neutrophils significantly shortened the mean time to death of the recipient *Cxcr2^−/−^* mice, a result consistent with a deleterious role for NE. In contrast, mice that received NE-deficient neutrophils showed a significantly longer survival. The fact that their survival was even longer than that of *Cxcr2^−/−^* mice that did not receive neutrophils may suggest that, in absence of elastase, neutrophils may play a protective role in melioidosis. This notion is in agreement with published works [21] and is supported by the observation that a single intranasal transfer of either wild type or NE-deficient neutrophils into C57BL/6 mice 18 hours p.i. significantly decreased organ bacterial burdens (supplementary figure S4A). However, multiple transfers of wild type neutrophils into infected *Elane^−/−^* mice increased lung tissue damage as well as bacteria burden (supplementary figure S4B, C). Collectively, these experiments support the notion that, in the early phase of the infection, and in limited number, neutrophils play a protective role against lung infection with *B. thailandensis*. In contrast, excessive and sustained neutrophil recruitment to the lung becomes deleterious due to the damaging action of NE and because of neutrophil's inability to restrain intracellular bacteria replication [6], [18]–[20].

**Figure 3:**
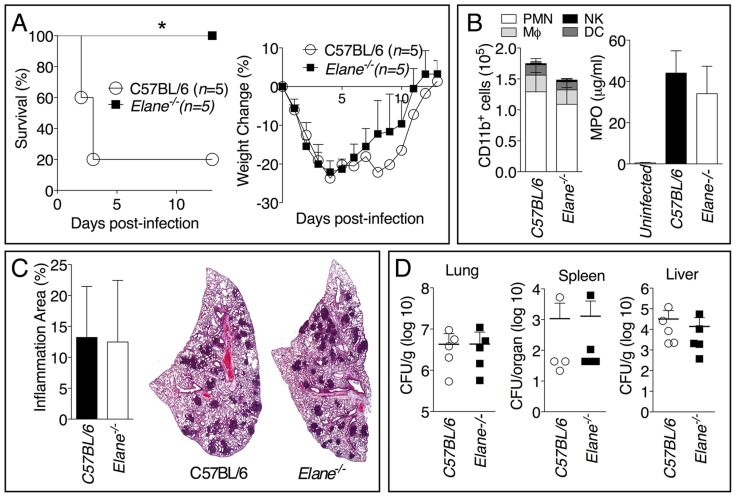
Neutrophil elastase reduces host tolerance to infection with *B. pseudomallei*. Mice (*n* = 5) were intranasaly infected with 400 CFU *B. pseudomallei* and their survival and weight were monitored (A). (B) Flow cytometric analysis of BAL cells and levels of MPO in BALF collected 72 h p.i. from mice (*n* = 5) infected as in A. (C) Representative lung sections stained with H&E and the total area of the inflammatory nodules was measured and expressed as percentage of the total lung lobe area (*n* = 5). (D) Tissue burdens of *B. pseudomallei* at 72 h p.i. Data are expressed as mean + S.D. *p<0.05. (A) log rank Kaplan-Meier test, (B–D) Mann-Whitney U test.

To further confirm our findings, we examined the role of NE during infection with the significantly more virulent agent *B. pseudomallei* and observed an identical scenario. The majority of C57BL/6J mice intranasaly infected with *B. pseudomallei* succumbed to infection whereas *Elane^−/−^* mice showed complete protection (fig. 3A). Both groups of mice lost weight to the same extent during the first 72–96 hours post infection, but only the *Elane^−/−^* mice (and a single C57B/L6J survivor) started to gain their weight back thereafter, suggesting that the initial wasting response to infection is not responsible for the susceptibility of C57BL/6J mice. Interestingly, weight loss in *Il-1r1^−/−^* mice infected with *B. pseudomallei* is much less prominent than in C57BL/6 mice (supplementary figure S5), suggesting that IL-1 deleterious role is not exclusively mediated by NE. Neutrophil recruitment and overall inflammation were comparable between C57BL/6J and *Elane^−/−^* mice (fig. 3B, C). Remarkably, C57BL/6J and *Elane^−/−^* mice had similar bacteria burdens in the lung, spleen, and liver (fig. 3D).

**Figure 4:**
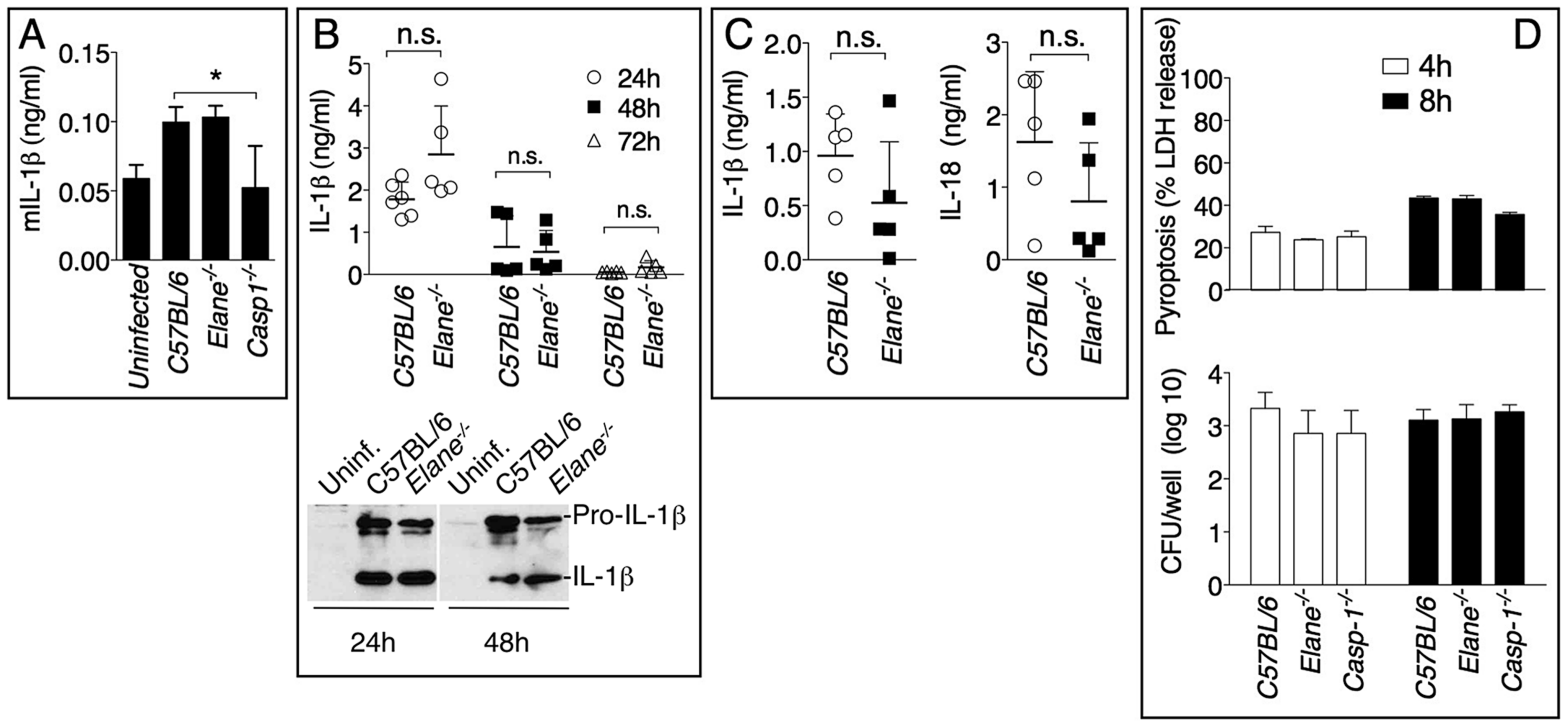
Role of elastase in IL-1β and IL-18 processing and pyroptosis. (A) Neutrophils were purified from bone marrow and infected in vitro with *B. thailandensis* 1∶100 m.o.i., and release of IL-1β was measured. (B, C) IL-1β and IL-18 were measured in BALF of mice infected with 5×10^5^ CFU *B. thailandensis* (B) or 400 CFU *B. pseudomallei* (C). (D) Pyropoptosis and intracellular bacteria replication in neutrophils infected in vitro as in (A). Data are expressed as mean + S.D. *p<0.05. (A, D) One way ANOVA Tukey Post-test, (B, C) Mann-Whitney U test. One representative experiment of three (A, B, D) or one (C) is shown.

**Figure 5:**
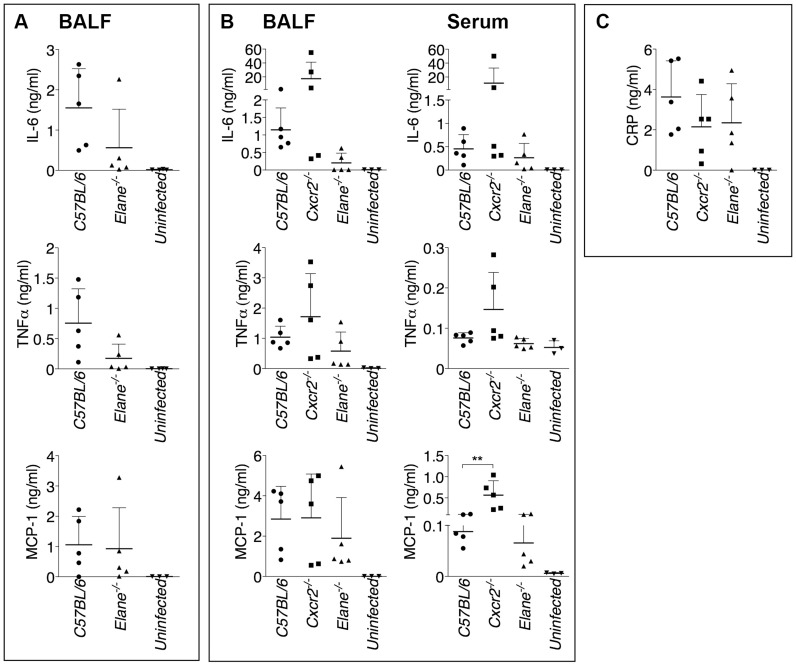
Inflammatory cytokines and acute phase proteins in BALF and sera of infected mice. Cytokines were measured in BALF of mice infected with 400 CFU *B. pseudomallei* (A) or in BALF and serum of mice infected with 5×10^5^ CFU *B. thailandensis* (B) 72 hours p.i. (C) Levels of C Reactive Protein in BALF of mice infected with 5×10^5^ CFU *B. thailandensis*. Data are expressed as mean + S.D. **p<0.01. Mann-Whitney U test. One representative experiment of two is shown.

It has been shown that during highly neutrophilic inflammation, proteases released by neutrophils, including NE, are able to process pro-IL-1β [22]–[24]. It is therefore conceivable that NE deficiency may protect mice from melioidosis because of decreased processing of IL-1β. However, C57BL/6J and *Elane^−/−^* neutrophils released equal amount of IL-1β when infected in vitro with *B. thailandensis* (fig. 4A) and mature IL-1β and IL-18 were present in equal amount in the BALF of *Elane^−/−^* or C57BL/6J mice infected with *B. thailandensis* or *B. pseudomallei* (fig. 4B, C). Absence of NE did not affect pyropoptosis (fig. 4D), ruling out modulation of this protective mechanism as the reason for the protective effect. The level of other pro-inflammatory cytokines and acute phase proteins in BALF or serum of *Elane^−/−^* mice were reduced, though not significantly, a possible reflection of increased release of endogenous alarmins (fig. 5).

### Neutrophil Elastase Causes Lung Tissue Damage and Increases Vascular Leakage

**Figure 6:**
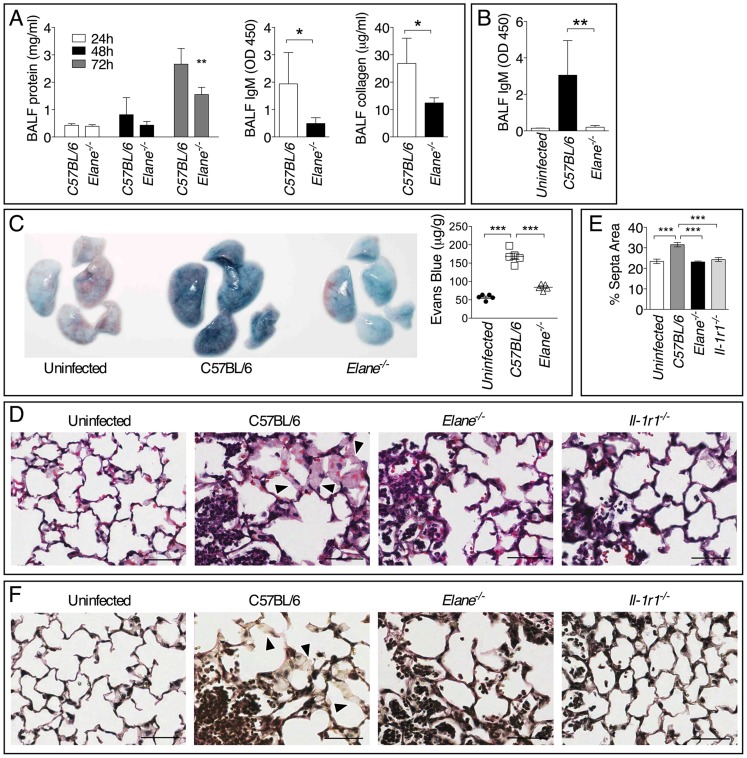
Neutrophil elastase causes lung tissue damage during infection with *Burkholderia* species. Total protein, IgM, and collagen were measured in the BALF collected at 72 h p.i. from mice infected with 5×10^5^ CFU *B. thailandensis* (*n* = 5) (A) or 400 CFU *B. pseudomallei* (*n* = 5) (B). (C) Extravascular leakage of intravenously administered Evans blue in mice infected with *B. thailandensis* as in (A) was measured 72 h p.i in the lung tissue (µg/g tissue). (D) Representative photographs of histological sections of lung tissues stained with H&E. Alveolar septa are significantly thicker in C57BL/6J than in *Elane^−/−^* or *Il-1r1^−/−^* mice (arrow heads). (E) Area of alveolar septa was measured by Image J software. (F) Verhoeff-van Gieson stain for elastic fibers. Reduced elastic fiber staining was observed in C57BL/6J mice but not in *Elane^−/−^* or *Il-1r1^−/−^* mice (arrow heads). Bar is 50 µm. Data are expressed as mean + S.D. *p<0.05, **p<0.01, ***p<0.001. Mann-Whitney U test. One representative experiment of three (A, C–F) or one (B) is shown.

Minimization of immune-mediated tissue damage is likely among the most effective strategies to increase host tolerance to infection. In agreement with this notion, significantly reduced tissue damage was measured in *Elane^−/−^* mice infected with *B. thailandensis* or *B. pseudomallei* compared to C57BL/6J mice as demonstrated by lower levels of IgM, collagen, and total protein in BALF (fig. 6A, B) and decreased extravascular leakage of intravenously administered Evans blue (fig. 6C). *Elane^−/−^* mice also had decreased BALF levels of surfactant protein D, another marker of tissue damage (supplementary figure S6) [25]–[27]. Reg3γ, a protein with antibacterial and regenerative functions released by epithelial cells in response to infection and damage [28], and the alarmin HMGB1 were also detected in higher amounts in the BALF of C57BL/6J than *Elane^−/−^* mice, consistent with the increased tissue damage. These findings were consistent with the conclusion that C57BL/6J mice experienced more tissue damage than the *Elane^−/−^* mice as a consequence of *Burkholderia* infection. Although the overall extent of inflammation was similar in infected C57BL/6J and *Elane^−/−^* mice (fig. 2 and 3), examination of H&E stained lung sections at high magnification revealed extensive hemorrhage indicating injury to the alveolar capillary endothelium and increased interstitial edema in C57BL/6J mice compared to *Elane^−/−^* or *Il-1r1^−/−^* mice. Image processing analysis was used to measure interstitial edema revealing that in C57BL/6J mice the alveolar septa are significantly thicker than in *Elane^−/−^* or *Il-1r1^−/−^* mice (fig. 6D, E arrow heads). Reduced elastic fiber staining was also observed in C57BL/6J mice but not in *Elane^−/−^* or *Il-1r1^−/−^* mice (fig. 6F), suggesting that NE-mediated degradation of alveolar basement membrane contributes to lung tissue damage.

### Bradykinin Contributes to NE-Mediated Lung Damage and Decreased Host Tolerance

**Figure 7:**
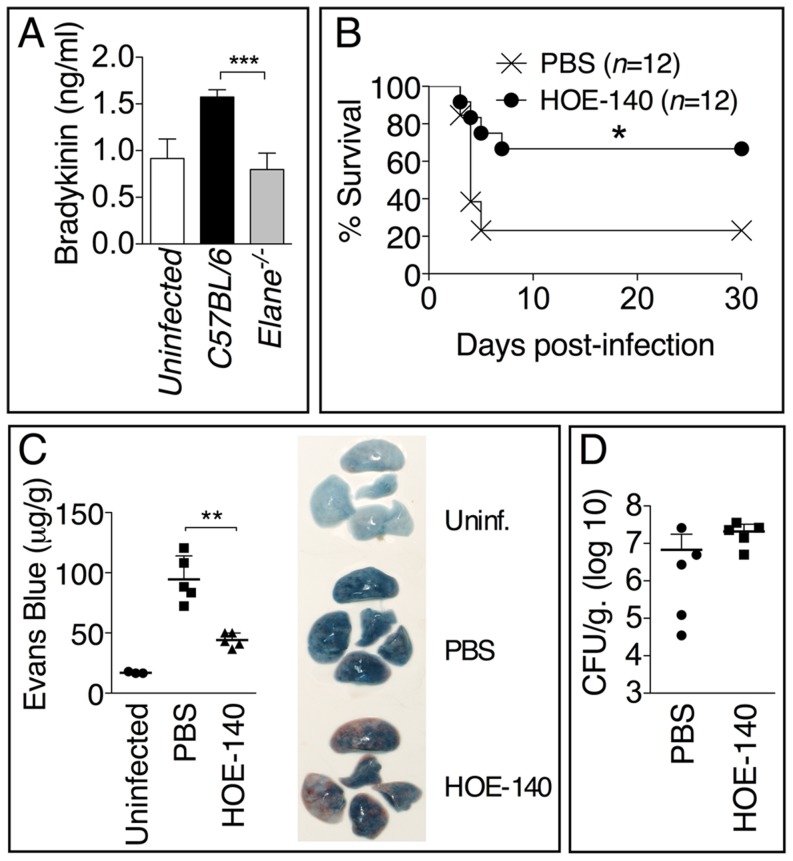
Bradykinin contributes to NE-mediated lung damage and decreased host tolerance. (A) Levels of bradykinin were measured 72 h p.i. in the BALF of mice (*n* = 5) infected with 5×10^5^ CFU *B. thailandensis*. (B) Mice (*n* = 12) infected with 5×10^5^ CFU *B. thailandensis* were administered bradykinin antagonist HOE-140 (10 µg i.n., daily) and their survival monitored. Results pooled from three experiments are shown. (C) Extravascular leakage of intravenously administered Evans blue (µg/g tissue) and bacteria burdens (D) were measured at 72 h p.i. Data are expressed as mean + S.D. *p<0.05, **p<0.01, ***p<0.001. (A, C, D) Mann-Whitney U test. (B) log rank Kaplan-Meier test. One representative experiment of three (A) or two (C, D) is shown.

Consistent with the decreased vascular leakage observed in *Elane^−/−^* mice, their BALF contained reduced levels of bradykinin (fig. 7A), a peptide that is critically involved in regulating vascular permeability and leakage. Treatment of infected mice with the bradykinin antagonist HOE-140 significantly increased mice survival (fig. 7B) and decreased vascular leakage (fig. 7C) but, more importantly, did not affect bacteria burden (fig. 7D). These results suggest that one of the mechanisms through which NE decreases tolerance to *Burkholderia* infections is by increasing generation of bradykinin and consequent vascular leakage.

## Discussion

The ability of a host to survive an infection depends on resistance mechanisms that eliminate the infectious agent, and on tolerance mechanisms that minimize the negative impact the infection has on the host's health without directly affecting pathogen burden [2], [3]. Our understanding of the mechanisms that regulate host tolerance to infection is still rudimentary. Early evidence that tolerance mechanisms existed in mammals was obtained by studying *Plasmodium*-infected mice with the observation that disease severity does not directly correlate with the parasite burden [29]. Using a similar mouse model of malaria, it has also been demonstrated that sickle hemoglobin, whose prevalence is elevated in areas of high malaria incidence, increases host tolerance to *Plasmodium* and, therefore, protects those who carry this mutation from severe malaria [30]. Sickle hemoglobin was shown to increase expression of heme oxygenase-1, an enzyme that converts the free heme into antioxidant molecules that are protective against oxidative damage caused by severe malaria. Heme oxygenase-1 also prevented a pathological immune response without affecting the parasite burden. A recent study has shown that promotion of tissue repair through administration of amphiregulin, an EGF family member that plays a role in maintaining lung homeostasis, can protect mice co-infected with influenza A virus and *Legionella pneumophila* [31]. The observed protective effect was not due to changes in pathogen burden, suggesting it affected tolerance rather than resistance mechanisms. Interestingly, in this model excessive inflammation and immunopathology did not appear to be the cause of morbidity and mortality. Analysis of resistance and tolerance mechanisms is often complicated because some host factors may control and participate simultaneously in both types of responses, as it has been proposed for the enzyme Chi3l1 during *Streptococcus pneumoniae* infection [32].

While neutrophils are critical for protection against several bacterial and fungal infections, their role in melioidosis remains unclear. We and others have shown that neutrophils are not very effective at containing *B. pseudomallei* infection and, in fact, may foster its systemic dissemination [6], [18]–[20]. Our results show that excessive PMN recruitment to the lung decrease host tolerance to melioidosis because of the damaging action of NE on lung tissue. However, our results also suggest that, in limited amounts and in absence of NE, neutrophils may play a protective role in melioidosis, in agreement with published work [21].

Our observation that NE plays a detrimental role in melioidosis is surprising because several reports have shown that this protease is crucially involved in defense against bacterial pathogens through various mechanisms including direct killing [33], degradation of virulence factors [34], or regulation of the inflammatory response by targeting cytokines, chemokines and their receptors [35], [36]. NE has also been shown to be crucial for deployment of neutrophil extracellular traps [37], an important antimicrobial effector mechanism [38]. NE-deficiency however, can also be beneficial during infections, as demonstrated by the fact that *Elane^−/−^* mice are protected from anthrax toxin [39] and that NE deficiency did not affect infection with *B. cepacia* complex [40], again suggesting that neutrophils and NE are not effective antimicrobial agents against *Burkholderia* species. Whether NE's effects on host's tolerance are due to direct action on the bacteria remains to be determined, but we were unable to obtain data supporting this possibility. It should be mentioned that any direct action of NE on bacteria would likely affect bacteria's proliferation/virulence, a scenario that contrasts with our observation that the protection conferred by NE deficiency is not associated with reduced bacteria burdens suggesting that NE is not targeting the bacterium but rather the host.

Although our results support a model where IL-1β is deleterious because of excessive recruitment of neutrophils and the subsequent NE release, it was surprising to find out that different defense mechanisms account for the protection in *Il-1r1^−/−^* mice compared to *Elane^−/−^* mice. Thus, despite bacteria burdens similar to those of C57BL/6J mice, *Elane^−/−^* mice have higher health indicating that the absence of NE increases tolerance rather than resistance to *B. thailandensis* and *B. pseudomallei*. In contrast, the higher health of *Il-1r1^−/−^* mice may involve both resistance and tolerance mechanisms; the decreased organ bacterial burdens observed in *Il-1r1^−/−^* mice is consistent with increased resistance while the decreased neutrophil influx, NE levels, and tissue damage are consistent with increased tolerance. Taken together, these results suggest that the deleterious role of IL-1β is not solely mediated by NE, even though NE absence is sufficient to confer protection by enhancing tolerance.

NE is known to cause host tissue damage leading to functional impairment of multiple organs, including the lungs [41], [42]. The deleterious action of NE has been best documented during experimental models of acute lung injury [43], [44] where this enzyme has been shown to inflict tissue damage by direct degradation of extracellular matrix components, adhesion molecules, antimicrobial and protective host factors such as the protein SPLUNC1 [45], mucins [46], surfactant protein A and D [47], and various cytokines. NE is a promiscuous protease, which suggests that multiple mechanisms may account for its negative impact on host tolerance to melioidosis. In our experiments we observed decreased collagen degradation in the BALF of *Elane^−/−^* mice compared to controls and decreased elastic fiber staining suggesting that NE may directly damage the lung connective tissues. The decreased level of bradykinin and the consequent diminished vascular leakage observed in *Elane^−/−^* mice suggests that generation of bradykinin may be an additional mechanism through which NE can exert its deleterious effect. NE and elastases of bacterial origin have been shown to be able to process kininogen and generate bradykinin [48], [49]. Alternatively, the damage caused by NE to the endothelium of alveolar capillaries may lead to activation of the contact system and generation of bradykinin [50].

Our attempts to obtain evidence that NE may degrade factors important for lung physiology, such as surfactants, were inconclusive. However, we found that proteins that exert antimicrobial and protective functions such as SP-D and Reg3γ were present in higher amounts in the BALF of C57BL/6J compared to *Elane^−/−^* mice. Induction of these factors is part of the physiologic response to lung damage and likely reflects the increased tissue damage inflicted by NE in C57BL/6J mice. Our study shows that neutrophil recruitment to the lung is not affected by NE deficiency, as previously shown [51]. Similarly, the overall extent of inflammation and the level of several proinflammatory cytokines, including IL-1β and IL-18, were not significantly different in presence or absence of NE. Production of mature IL-1β by infected *Elane^−/−^* mice or in vitro-infected *Elane^−/−^* neutrophils was comparable to the controls suggesting that during melioidosis the majority of IL-1β is generated in a caspase-1-dependent fashion, as we and others previously showed [6], [52].

Considering that defense mechanisms such as IL-1 and NE that are protective in a majority of bacterial and viral infections become deleterious in melioidosis clearly sets *Burkholderia* apart from most Gram-negative bacteria. This raises the possibility that other infectious models where excessive inflammation is known to be deleterious [53], [54] can become useful to separate and identify the relative contribution of resistance and tolerance to host survival. Bacterially expressed elastases have been shown to act as virulence factors [55]. Future studies should examine whether *Burkholderia* species adopt this strategy.

In conclusion, our study reveals that NE is a host effector mechanism that can negatively impact the host's tolerance to infection with *Burkholderia* species by causing lung tissue damage and increasing bradykinin-mediated alveolar microvascular permeability. Counteracting NE activity through the use of clinically tested protease inhibitors has proven to be beneficial in a number of human inflammatory conditions and mouse models [56], [57]. Our study shows that this may be an effective therapeutic intervention for melioidosis.

## Materials and Methods

### Ethics Statement

All the animal experiments described in the present study were conducted in strict accordance with the recommendations in the *Guide for the Care and Use of Laboratory Animals* of the National Institutes of Health. All animal studies were conducted under protocols approved by the Rosalind Franklin University of Medicine and Science Institutional Animal Care and Use Committee (IACUC) (protocol # B12-07), and the University of Tennessee Health Science Center IACUC (protocol #1854). All efforts were made to minimize suffering and ensure the highest ethical and humane standards.

### Mice

C57BL/6J, *Il-1r1^−/−^*, *Il-18^−/−^*, *Elane^−/−^*, *Mmp12^−/−^*, *Nos2^−/−^*, *gp91^phox−/−^*, and *Cxcr2^+/−^* mice were purchased from Jackson lab. *Il-1a^−/−^*and *Il-1a^−/−^/Il-1b^−/−^* were from I. Iwakura, *Il-1b^−/−^* from D. Chaplin, *Asc^−/−^*, *Nlrp3* ^−/−^, *Nlrc4^−/−^* from V. Dixit, and *Casp1* ^−/−^ from F. Sutterwala. *Il-18^−/−^-Il-1r1^−/−^* double deficient mice (DKO) were obtained by crossing the parental single knock-out mice. *Cxcr2^−/−^* mice were obtained by crossing heterozygotes. All mouse strains were on C57BL/6J genetic background and were bred under specific pathogen-free conditions. Age-(8–12 weeks old) and sex-matched animals were used in all experiments. Experimental groups were composed of at least 5 mice, unless stated otherwise.

### Bacteria Strains and Intranasal Infections

*B. thailandensis* E64 was obtained from ATCC. *B. pseudomallei* 1026b strain is a clinical virulent isolate. Bacteria were grown in Luria broth to mid-logarithmic phase, their titer was determined by plating serial dilutions on LB agar, and stocks were maintained frozen at −80°C in 20% glycerol. For infections, frozen stocks were diluted in sterile PBS to the desired titer. Mice were anesthetized using isoflurane and the infectious doses were applied to the nares in 50 µl total volume. All work with *B. pseudomallei* was performed under biosafety level-3 (BSL3/ABSL3) containment according to policies and standard operating procedures approved via the UTHSC Committee on Biocontainment and Restricted Entities, a subcommittee of the UTHSC Institutional Biosafety Committee. The UTHSC has been approved for select agent work by the Centers for Disease Control.

### Determination of Bacteria Growth in Organs

Organs aseptically collected were weighted and homogenized in 1 ml PBS using 1 mm zirconium beads and the Mini16 bead beater. Serial dilutions were plated as described above on LB agar plates containing Streptomycin (100 µg/ml).

### BALF Collection, Cytokines, IgM, Collagen, Myeloperoxidase, and Elastase Measurements

BALF were collected from euthanized mice by intratracheal injection and aspiration of 1 ml PBS. Cytokines levels in tissue culture conditioned supernatants, BALF, or sera were measured by ELISA using the following kits: mIL-1β , mIL-6, mTNFα, mMCP (eBioscience), mIL-18 (MBL Nagoya, Japan), mCRP (Abcam), Bradykinin (Enzo), IgM (Southern Biotech). Collagen in BALF was measured using the hydroxyproline assay kit (Condrex Inc). Myeloperoxidase or elastase activity were measured in lung homogenates using the MPO fluorometric detection kit (Enzo) or as previously described [58]. In Figure 3B, MPO was measured in the BALF by ELISA (Hycult Biotech).

### Evans Blue

Two hours before euthanasia, infected mice were intravenously injected with 0.312 mg Evans Blue in a volume of 0.2 ml PBS. After BALF collection, lungs were perfused with PBS, incubated in formamide for 18 hours at 37°C to extract Evans blue, and absorbance was measured at 620 nm.

### Flow Cytometry

Cells obtained from BALF were counted and stained with CD11b, CD11c, F4/80, Ly6G, NK1.1 and acquired with a LSRII BD flow cytometer. Analysis was performed with FACS DIVA software.

### Histology and Measurement of Area of Inflammatory Foci and Interstitial Edema

For lung fixation, the trachea was cannulated and the lung was inflated by instillation of 4% paraformaldehyde at the pressure of 25 cm/H_2_O. Formalin-fixed paraffin-embedded lung sections were stained with H&E or Verhoeff-van Gieson and scanned using the Aperio Scanscope XT. The Aperio ImageScope software was used to quantitate the area of the inflammatory foci compared to the total lung lobe area. ImageJ software was used to determine interstitial edema as the area of the alveolar septa. For each lung sections, five random fields (20× magnification, 793×794 pixels∼160,000 µm^2^) at that did not contain inflammatory foci or bronchioles or blood vessels were selected. Using “magic wand” tool of ImageJ, the empty alveolar spaces were deleted; the threshold color was adjusted to select only the eosin staining (septa); the image was converted to 8-bit and the threshold adjusted. ImageJ was used to measure the percentage of eosin stained area over the total area in each image. Results from lungs of 5 animals (H&E).

### Western Blot

BALF were separated by SDS-PAGE, transferred to PVDF membranes, and probed with rabbit anti-mSP-D (Abcam), rabbit anti-HMGB1 (Abcam), rabbit anti-mReg3γ (Abgent), or goat anti-mIL-1β (R&D Systems).

### Mouse Neutrophil Isolation and Adoptive Transfer

Neutrophils were isolated from bone marrow of C57BL/6J or *Elane^−/−^* mice using Miltenyi Ly6G microbeads. For adoptive transfer, *Cxcr2^−/−^* mice were intranasaly infected with 2×10^5^ CFU *B. thailandensis* and 1.5×10^6^ neutrophils of either genotype were transferred into recipient mice 24 and 96 hr after infection by retro-orbital intravenous injection in a volume of 0.15 ml.

### Pyroptosis and Intracellular Bacteria Growth

Release of LDH in tissue culture media, a reflection of pyroptosis, was measured using the Roche Cytotox detection kit. Neutrophils (5×10^5^ cells) were plated in 24 well plates. Bacteria were added to the cell culture and the plates were centrifuged at 1500 rpm for 10 minutes to maximize and synchronize infection and incubated for 30 minutes at 37°C. Cells were washed with PBS to remove extracellular bacteria and medium containing kanamycin (200 µg/ml) was added to inhibit extracellular bacteria growth. Media were collected at 4 and 8 hours post infection for LDH measurement. Cells were lysed in PBS-2% saponin-15% BSA and serial dilutions of the lysates were plated on LB agar plates containing streptomycin (100 µg/ml) using the Eddy Jet Spiral Plater (Neutec). Bacterial colonies were counted 48 hours later using the Flash & Grow Automated Bacterial Colony Counter (Neutec).

### Statistical Analysis

All data were expressed as mean + S.D. Survival curves were compared using the log rank Kaplan-Meier test. Mann-Whitney U test or One way ANOVA Tukey Post-test were used for analysis of the rest of data as specified in the figure legends. Significance was set at *p*<0.05.

### Accession Numbers

Mouse Elane- Q3UP87

Mouse NOS2- P29477

Mouse gp91phox- Q61093

Mouse CXCR2- P35343

Mouse MMP12- P34960

Mouse IL1alpha- P01582

Mouse Il-1beta- p10749

Mouse IL-1RI- P13504

Mouse IL-18- P70380

Mouse NLRP3- Q8R4B8

Mouse NLRC4- Q3UP24

Mouse ASC- Q9EPB4

Mouse Casp-1- P29452

Mouse SP-D- P50404

Mouse Reg3gamma- O09049

Mouse HMGB1- P63158

## Supporting Information

Figure S1 **Role of inflammasome components in the response to** ***B. thailandensis*** **infection.** (A, B) BMDM were infected in vitro with *B. thailandensis* at 1∶100 m.o.i. Release of IL-1β (A), and induction of pyropoptosis and intracellular bacteria replication (B) were measured at the indicated time points. (C, D) Mice were infected intranasaly with 5×10^5^ CFU *B. thailandensis* and IL-1β in BALF and lung bacterial burdens were determined 48 h p.i. Data are expressed as mean + S.D. *p<0.05, **p<0.01, ***p<0.001. One way ANOVA Tukey Post-test (A, B). Mann-Whitney U test (C, D). One representative experiment of three is shown.
(DOCX)

Figure S2 **Presence of neutrophils in alveolar spaces or lung parenchyma.** Neutrophils numbers were measured by flow cytometry in BALF or total lung digested with collagenase/DNase from the indicated mouse strains infected intranasaly with 5×10^5^ CFU *B. thailandensis* at 72 h p.i. (*n* = 5). Data are expressed as mean + S.D. Mann-Whitney U test.
(DOCX)

Figure S3 **Role of MMP12 in melioidosis.** C57BL/6J or *Mmp12^−/−^* mice (*n* = 5) were infected intranasaly with 5×10^5^ CFU *B. thailandensis* and their survival was monitored. One representative experiment of two is shown.
(DOCX)

Figure S4 **A single administration of neutrophils is protective but multiple administrations are deleterious.** (A) *C57BL/6* mice (*n* = 4) were intranasaly infected with 5×10^5^ CFU *B. thailandensis*. C57BL/6J or *Elane^−/−^* neutrophils (2.5×10^6^) were administered intranasaly 18 h p.i and bacteria burden were measured at 72 h p.i. (B, C) *Elane^−/−^* mice were intranasaly infected with 5×10^5^ CFU *B. thailandensis*. Wild type neutrophils (6×10^6^) were administered intranasaly 48 h and 62 h p.i. Extravascular leakage of intravenously administered Evans blue (µg/g tissue) (B) and bacteria burdens were measured at 72 h p.i. Data are expressed as mean + S.D. *p<0.05, **p<0.01, ***p<0.001. Mann-Whitney U test. One representative experiment of two is shown.
(DOCX)

Figure S5 **Decreased weight loss in** ***Il-1r^−/−^*** **mice infected with** ***B. pseudomallei*** **.** Mice (*n* = 5) were intranasaly infected with 400 CFU *B. pseudomallei* and their weight was monitored. Data are expressed as mean + S.D. *p<0.05. Mann-Whitney U test. One representative experiment of two is shown.
(DOCX)

Figure S6 **Decreased tissue damage in** ***Elane^−/−^*** **mice infected with** ***B. thailandensis*** **.** Western blot analysis of SP-D, Reg3γ, and HMGB1 in BALF of infected mice 72 h p.i. One representative experiment of four is shown.
(DOCX)
