# Supplementary figures and images for: Neutrophil Elastase Causes Tissue Damage That Decreases Host Tolerance to Lung Infection with Burkholderia Species
Source: PLoS Pathog. 2014 Aug 28;10(8):e1004327. doi: 10.1371/journal.ppat.1004327 (PMC4148436; doi:10.1371/journal.ppat.1004327)

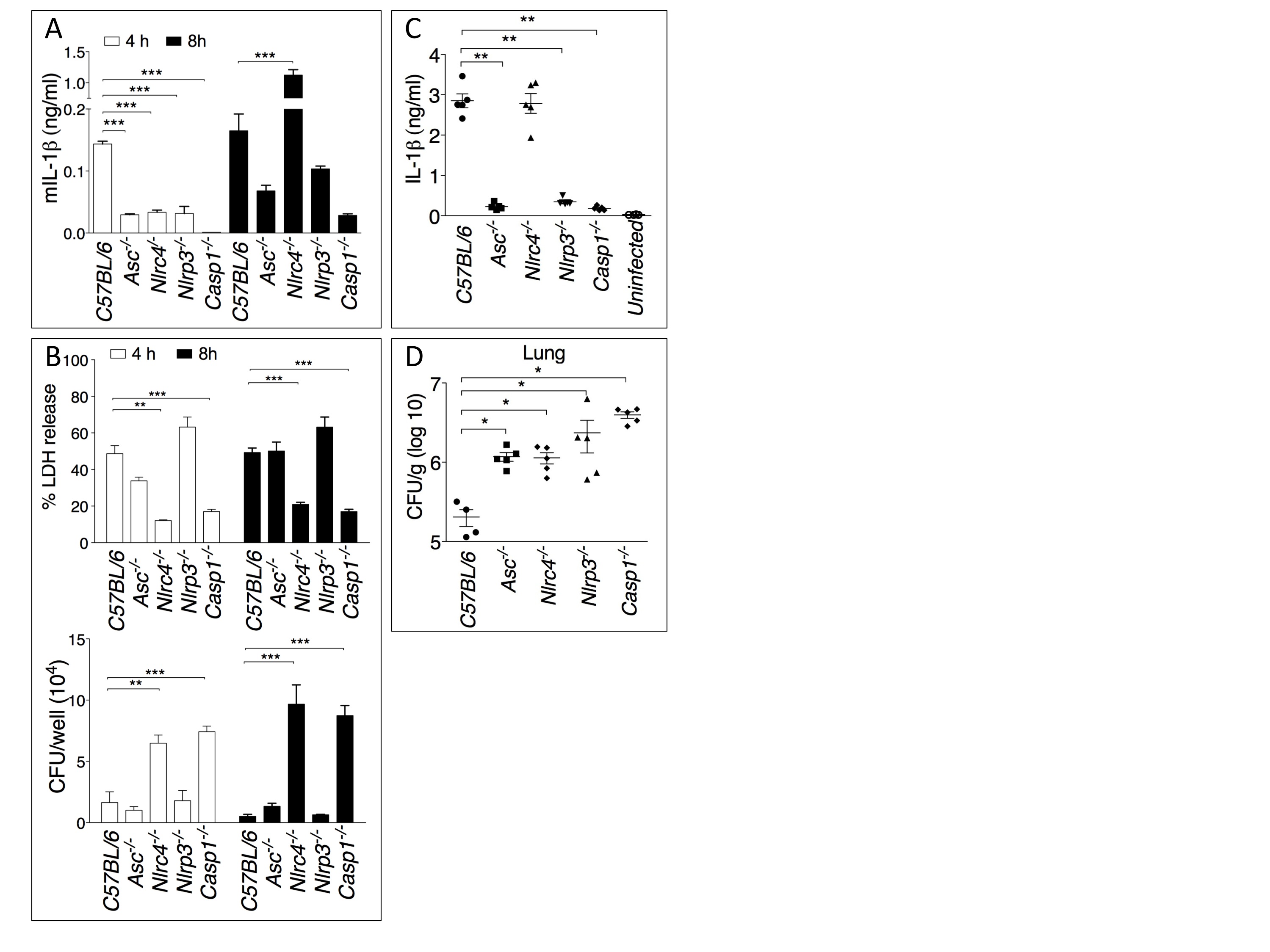


**Supplementary Figure S1**

Supplement: Figure S1 — Role of inflammasome components in the response to B. thailandensis infection. (A, B) BMDM were infected in vitro with B. thailandensis at 1∶100 m.o.i. Release of IL-1β (A), and induction of pyropoptosis and intracellular bacteria replication (B) were measured at the indicated time points. (C, D) Mice were infected intranasaly with 5×10^5^ CFU B. thailandensis and IL-1β in BALF and lung bacterial burdens were determined 48 h p.i. Data are expressed as mean + S.D. *p<0.05, **p<0.01, ***p<0.001. One way ANOVA Tukey Post-test (A, B). Mann-Whitney U test (C, D). One representative experiment of three is shown. (DOCX) [file ppat.1004327.s001.docx]

**
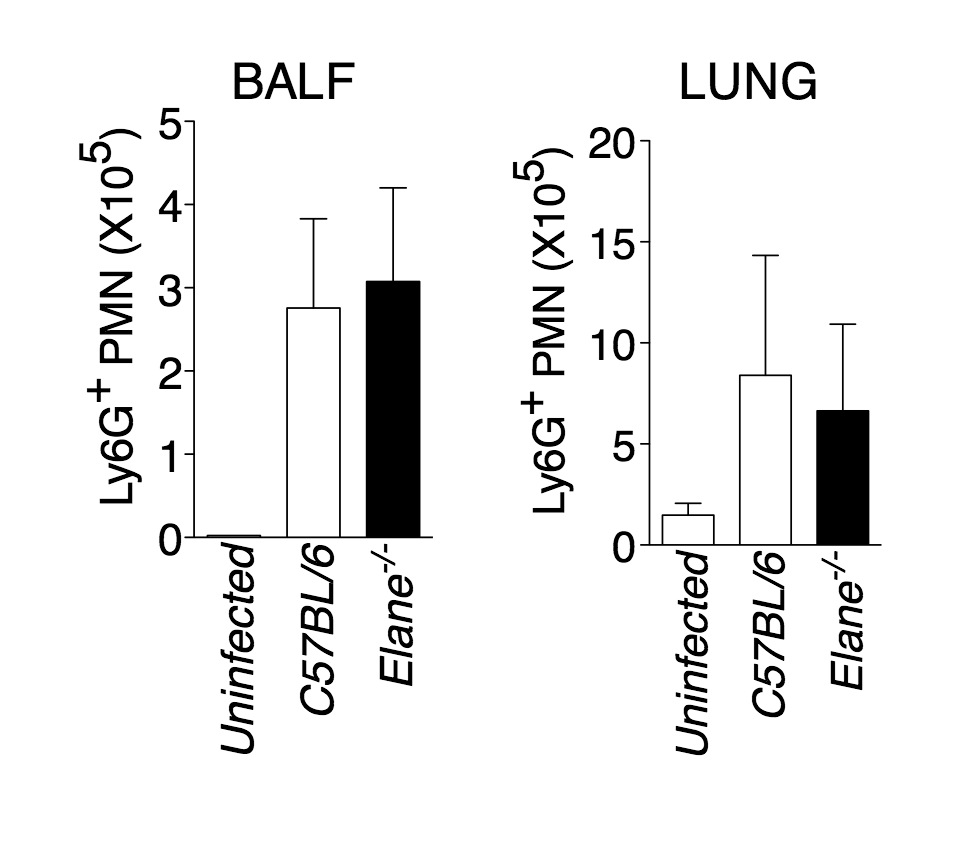
**

**Supplementary figure S2**

Supplement: Figure S2 — Presence of neutrophils in alveolar spaces or lung parenchyma. Neutrophils numbers were measured by flow cytometry in BALF or total lung digested with collagenase/DNase from the indicated mouse strains infected intranasaly with 5×10^5^ CFU B. thailandensis at 72 h p.i. (n = 5). Data are expressed as mean + S.D. Mann-Whitney U test. (DOCX) [file ppat.1004327.s002.docx]

**
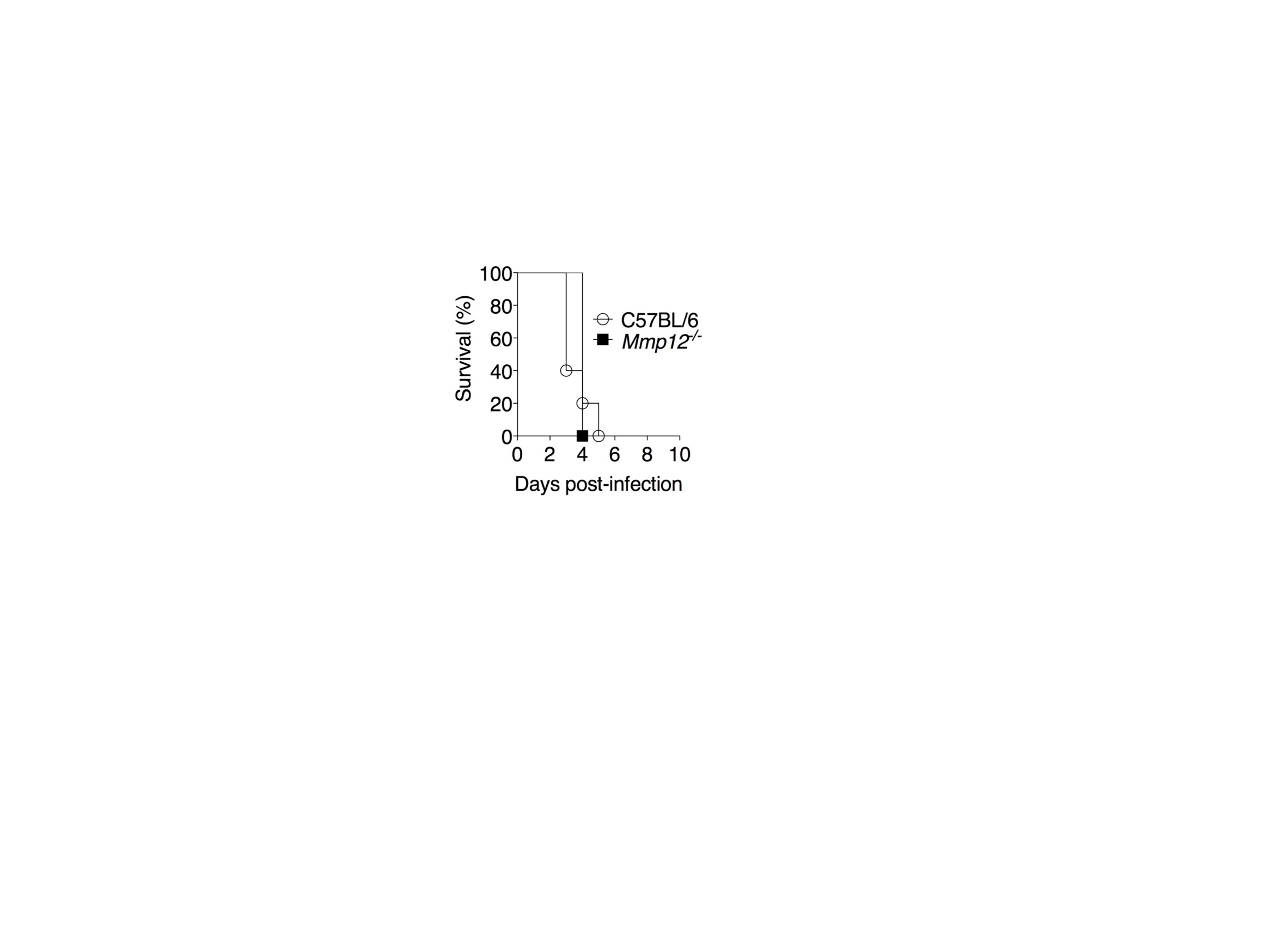
**

**Supplementary figure S3**

Supplement: Figure S3 — Role of MMP12 in melioidosis. C57BL/6J or Mmp12−/− mice (n = 5) were infected intranasaly with 5×10^5^ CFU B. thailandensis and their survival was monitored. One representative experiment of two is shown. (DOCX) [file ppat.1004327.s003.docx]

**
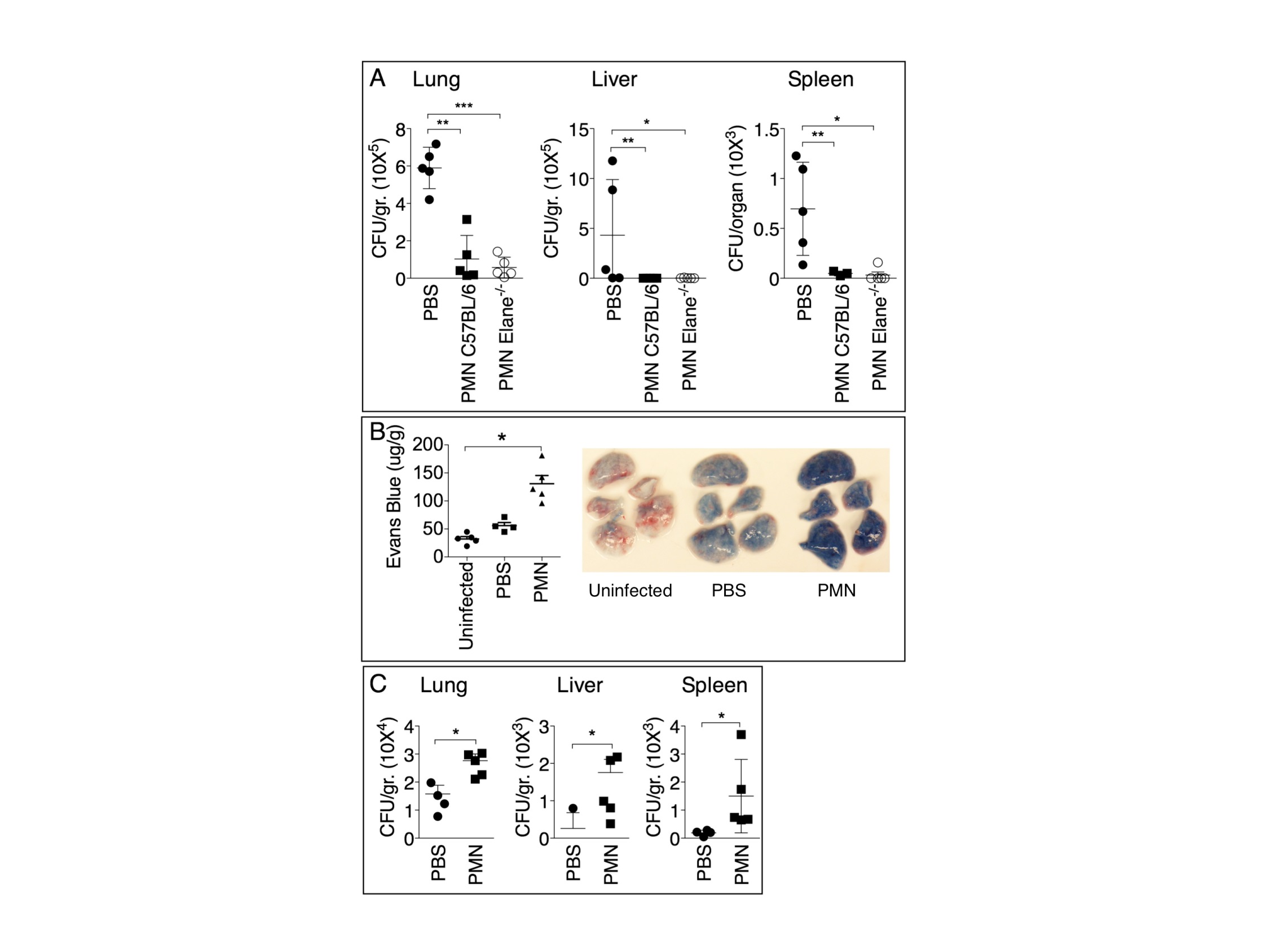
**

**Supplementary Figure S4**

Supplement: Figure S4 — A single administration of neutrophils is protective but multiple administrations are deleterious. (A) C57BL/6 mice (n = 4) were intranasaly infected with 5×10^5^ CFU B. thailandensis. C57BL/6J or Elane−/− neutrophils (2.5×10^6^) were administered intranasaly 18 h p.i and bacteria burden were measured at 72 h p.i. (B, C) Elane−/− mice were intranasaly infected with 5×10^5^ CFU B. thailandensis. Wild type neutrophils (6×10^6^) were administered intranasaly 48 h and 62 h p.i. Extravascular leakage of intravenously administered Evans blue (µg/g tissue) (B) and bacteria burdens were measured at 72 h p.i. Data are expressed as mean + S.D. *p<0.05, **p<0.01, ***p<0.001. Mann-Whitney U test. One representative experiment of two is shown. (DOCX) [file ppat.1004327.s004.docx]

**
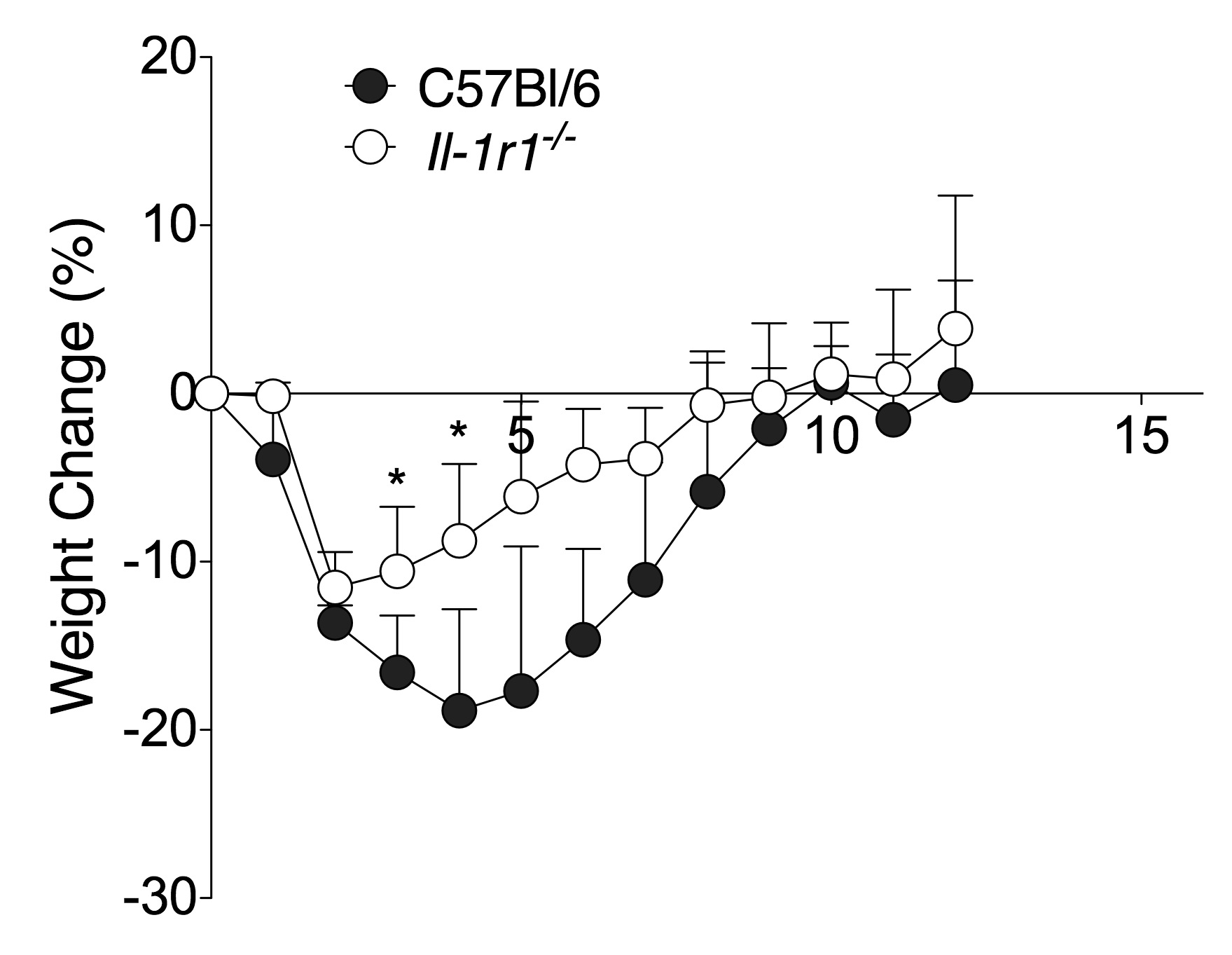
**

**Supplementary figure S5**

Supplement: Figure S5 — Decreased weight loss in Il-1r−/− mice infected with B. pseudomallei . Mice (n = 5) were intranasaly infected with 400 CFU B. pseudomallei and their weight was monitored. Data are expressed as mean + S.D. *p<0.05. Mann-Whitney U test. One representative experiment of two is shown. (DOCX) [file ppat.1004327.s005.docx]

**
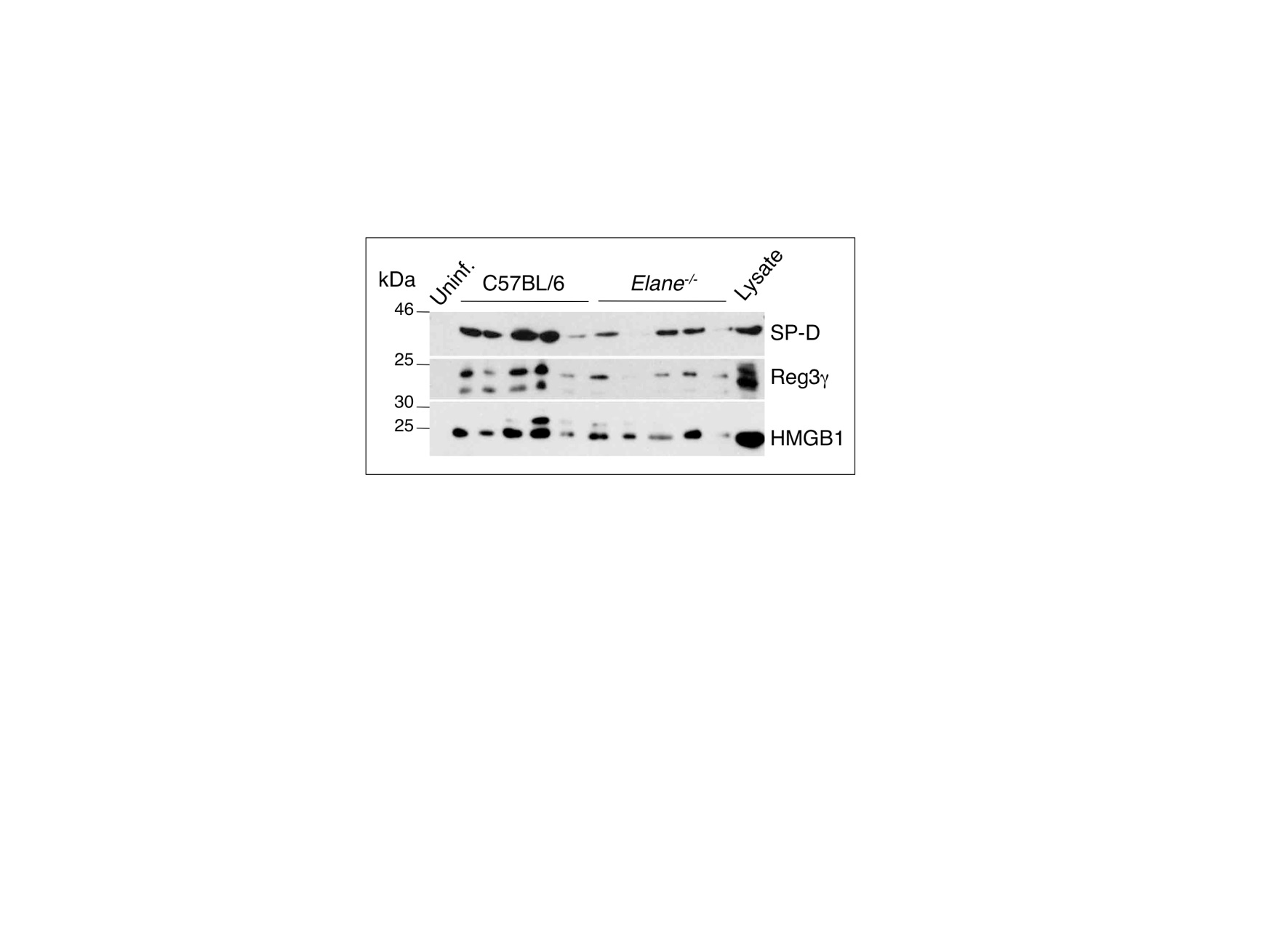
**

**Supplementary Figure S6**

Supplement: Figure S6 — Decreased tissue damage in Elane−/− mice infected with B. thailandensis . Western blot analysis of SP-D, Reg3γ, and HMGB1 in BALF of infected mice 72 h p.i. One representative experiment of four is shown. (DOCX) [file ppat.1004327.s006.docx]
